# Expression of IL-8, IL-6 and IL-1β in Tears as a Main Characteristic of the Immune Response in Human Microbial Keratitis

**DOI:** 10.3390/ijms16034850

**Published:** 2015-03-03

**Authors:** Concepcion Santacruz, Marisela Linares, Yonathan Garfias, Luisa M. Loustaunau, Lenin Pavon, Sonia Mayra Perez-Tapia, Maria C. Jimenez-Martinez

**Affiliations:** 1 Cornea and Refractive Surgery Department, and Research Unit, Institute of Ophthalmology “Conde de Valenciana Foundation”, Mexico 06800, DF, Mexico; E-Mails: csantivaldes@hotmail.com (C.S.); marisela.linares@yahoo.com.mx (M.L.); luisalousta@hotmail.com (L.M.L.); 2 Department of Biochemistry, Faculty of Medicine, National Autonomous University of Mexico, P.O. Box 70159, Mexico 04510, DF, Mexico; E-Mail: yogarfias@bq.unam.mx; 3 Department of Psychoimmunology, National Institute of Psychiatry “Ramón de la Fuente”, Calzada México-Xochimilco 101, Col. San Lorenzo Huipulco, Tlalpan, Mexico City 14370, DF, Mexico; E-Mail: lkuriaki@gmail.com; 4 Unit of R&D in Bioprocesses (UDIBI), Department of Immunology, National School of Biological Sciences, National Polytechnic Institute, Mexico 11340, DF, Mexico; E-Mail: smpt.2011@hotmail.com

**Keywords:** microbial keratitis, cytokines, IL-1β, IL-6, IL-8, NK cells, human

## Abstract

Corneal infections are frequent and potentially vision-threatening diseases, and despite the significance of the immunological response in animal models of microbial keratitis (MK), it remains unclear in humans. The aim of this study was to describe the cytokine profile of tears in patients with MK. Characteristics of ocular lesions such as size of the epithelial defect, stromal infiltration, and hypopyon were analyzed. Immunological evaluation included determination of interleukine (IL)-1β, IL-6, IL-8, IL-10, IL-12 and tumor necrosis factor (TNF)-α in tear samples obtained from infected eyes of 28 patients with MK and compared with their contralateral non-infected eyes. Additionally, frequency of CD4^+^, CD8^+^, CD19^+^ and CD3^−^CD56^+^ cells was also determined in peripheral blood mononuclear cells in patients with MK, and compared with 48 healthy controls. Non-significant differences were observed in the size of the epithelial defect, stromal infiltration, and hypopyon. Nevertheless, we found an immunological profile apparently related to MK etiology. IL-8 > IL-6 in patients with bacterial keratitis; IL-8 > IL-6 > IL-1β and increased frequency of circulating CD3^−^CD56^+^ NK cells in patients with gram-negative keratitis; and IL-8 = IL-6 > IL-1β in patients with fungal keratitis. Characterization of tear cytokines from patients with MK could aid our understanding of the immune pathophysiological mechanisms underlying corneal damage in humans.

## 1. Introduction

Corneal infections are frequent and potentially vision-threatening diseases; Prompt diagnosis and optimal treatment are necessary to prevent devastating outcomes. The World Health Organization and Vision 2020 have recognized that corneal diseases are the second leading cause of blindness after cataract and a major cause of preventable blindness in the developing world [1,2]. Many studies have emphasized the importance of determining the epidemiological profile and performing microbial identification with regard to describing the predisposing factors to develop microbial keratitis (MK) [3,4,5,6]. Factors such as trauma, contact lens wear, dry eye, ocular surface disorders and immunosuppression may alter the immune defense mechanisms of the outer eye, and permit microorganisms to invade the cornea [7]. Although MK is a rare condition in the absence of risk factors, 1.5–2 millions of new cases arise globally, and its presentation is considered a silent epidemic [8]. This is likely to be the case in countries, such as India, where the incidence of MK is ten times more frequent compared to the United States [9].

The most common pathogens recognized in MK are bacteria (*Staphylococcus aureus*, and *Streptococcus pneumoniae*), and fungi (*Aspergillus* spp., *Fusarium* spp., and *Candida* spp.) [10,11,12]. These microorganisms alter the protective physical barrier at the ocular surface, invading the cornea and initiating an immune/inflammatory response. The immunological mechanisms of ocular damage in MK have been studied in several animal models, wherein IL-1β has been proposed to mediate corneal injury induced by fungal and gram-negative bacteria [13,14]. Studies on human epithelial-derived cells and gram-negative bacteria have suggested that IL-6 and IL-8 are cytokines involved in immune-induced corneal damage [15]. Recently, Vasanthi *et al.*, observed increased concentrations of IL-6 and IL-8 in tear samples from patients with fungal keratitis [16]. Likewise, Yamaguchi *et al.*, identified the presence in tears of the inflammatory cytokines IL-1β, IL-6, and IL-8. Interestingly, they reported that increased levels of cytokines, correlated with corneal changes in unilateral infectious keratitis [17]. This evidence suggests that there is a certain profile of inflammatory cytokines based upon the infectious agent involved; however, little is known about the systemic response in patients with MK that could contribute to the immunopathogenesis in corneal ulcers. Therefore, the present study identifies systemic participation of peripheral blood mononuclear cells, together with pro-inflammatory cytokines in human tears.

## 2. Results

### 2.1. Demographics, and Relevant Medical History of Patients with Microbial Keratitis

The mean age of patients with fungal keratitis was 49 ± 19 years (*n* = 14), 65 ± 19 years for those with gram-positive keratitis (*n* = 8), and 51 ± 19 years for patients with gram-negative keratitis (*n* = 6). Microbial identification was performed in all cases. The most frequent causative microorganism of fungal keratitis was *Fussarium* spp.; Gram-positive bacterial keratitis was caused by *Streptococcus* spp.; While gram-negative bacterial keratitis was originated by *Pseudomonas* spp. (See Table 1). All patients included in this study had a relevant medical history (risk factors) that was related to microbial keratitis (See Table 1, and Tables S1–S3).

**Table 1:** Demographics and relevant medical history of patients with microbial keratitis.

Characteristics of Patients	Patients with Fungal Keratitis (*n* = 14)	Patients with Gram-Positive Bacterial Keratitis (*n* = 8)	Patients with Gram-Negative Bacterial Keratitis (*n* = 6)	Healthy Volunteers (*n* = 48)	*p* Value
Males/Females	10/4	3/5	3/3	24/24	–
Age (mean ± SD, years)	49 ± 19	65 ± 19	51 ± 19	52 ± 19	NS
Microbiological diagnosis (number of patients)	*Cephalosporium* spp. (1) *Curvularia* spp. (1) *Candida albicans* (1) *Trichoderma* spp. (1) *Aspergillus* spp. (1) *Fusarium* spp. (9)	*S. pneumonie* (3) *Streptococcus* spp. (2) *Micrococcus varians* (1) *S. pneumonie* and *S. epidermidis* (2)	*K. pneumonie* (1) *K. ozanae* (1) *Pseudomonas* sp. (3) *S. marcescens* (1)	–	–
Relevant medical history	Diabetes (4) Eye trauma (3) Diabetes and eye trauma (1) Alcoholism (1) Lens wear (1) None (4)	Diabetes (2) Previous herpes infection (2) Eye trauma (1) Congenital glaucoma (1) Rheumatoid arthritis (2)	Penetrating keratoplasty (1) Cosmetic lens/Lens wear (2) Diabetes (2) Drug user (1)	–	–

SD—Standard deviation; NS—Not significant.

### 2.2. Ocular Findings of Patients with Microbial Keratitis

**Figure 1:**
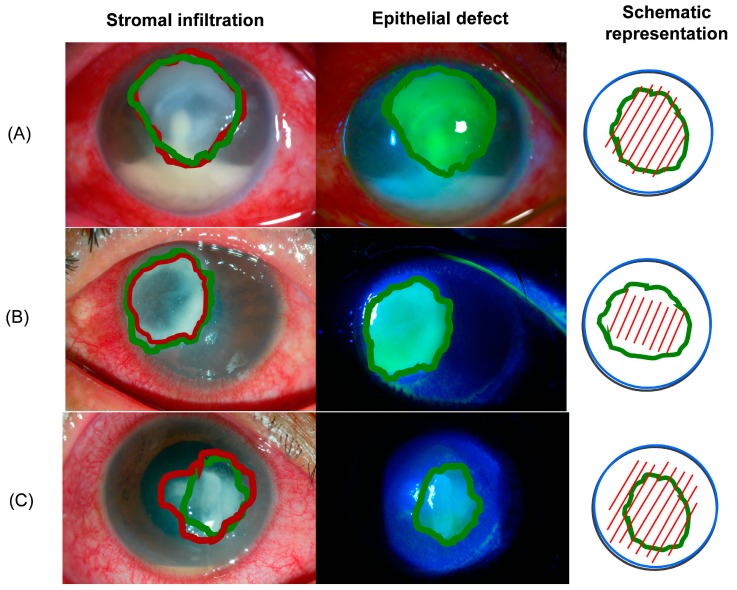
Representative clinical photographs of human microbial keratitis, Patient eye with Gram-positive bacterial keratitis (**A**); important conjunctival hyperemia with superior corneal infiltration (red) similar in size to the epithelial defect (green), a central fountain-like deposition linked to hypopyon, and creamy reaction in the anterior chamber. The causative microorganism was *S. pneumoniae*; Patient eye with gram-negative bacterial keratitis (**B**); stromal infiltration (red) apparently surrounded at the edge by the epithelial defect (green). The causative agent was *Pseudomonas* spp.; Patient eye with fungal keratitis; (**C**) a central epithelial defect (green) is seen with stromal infiltration, (red) and a typical feathery appearance surrounding the lesion. No lysis is observed, and the hypopyon has a white-creamy appearance. The causative agent was *Fusarium* spp. Schematic representation according with the size of the epithelial defect (green) and stromal infiltration (red) is proposed in the third column. Epithelial defects were observed under slit-lamp illumination with cobalt blue filter. See also Table 2.

Size of the epithelial defect, stromal infiltration, and the percentage of hypopyon were documented in all groups of patients. Although none of these clinical data differed significantly between groups, a careful analysis of the size of the epithelial defect, and the stromal infiltration showed clinical relevance. The results are depicted in Table 2, and representative clinical photographs are presented in Figure 1.

**Table 2:** Ocular findings in patients with microbial keratitis.

Ocular Findings	Fungal Keratitis (*n* = 14)	Gram-Positive Bacterial Keratitis (*n* = 8)	Gram-Negative Bacterial Keratitis (*n* = 6)	*p* Value
Epithelial defect: Horizontal diameter (mm)	5.3 ± 1.9	4.8 ± 1.9	5.6 ± 1.8	NS between any groups of patients.
Epithelial defect: Vertical diameter (mm)	5.7 ± 2.2	5.5 ± 2.3	5 ± 2.4	NS between any groups of patients.
Stromal lesion: Horizontal infiltration diameter (mm)	5.7 ± 2.4	4.9 ± 2.1	5 ± 2.4	NS between any groups of patients.
Stromal lesion: Vertical infiltration diameter (mm)	6.0 ± 2.6	5.6 ± 2.3	4.3 ± 1.5	NS between any groups of patients.
Hypopyon (percentage)	21 ± 22	37.5 ± 31	7.6 ± 8.6	NS between any groups of patients.

NS—Not significant; Mean ± SD (Standard deviation).

### 2.3. Frequency of CD3^−^CD56^+^ NK Cells Is Increased in Peripheral Blood Mononuclear Cells from Patients with Gram-Negative Bacterial Keratitis

**Figure 2:**
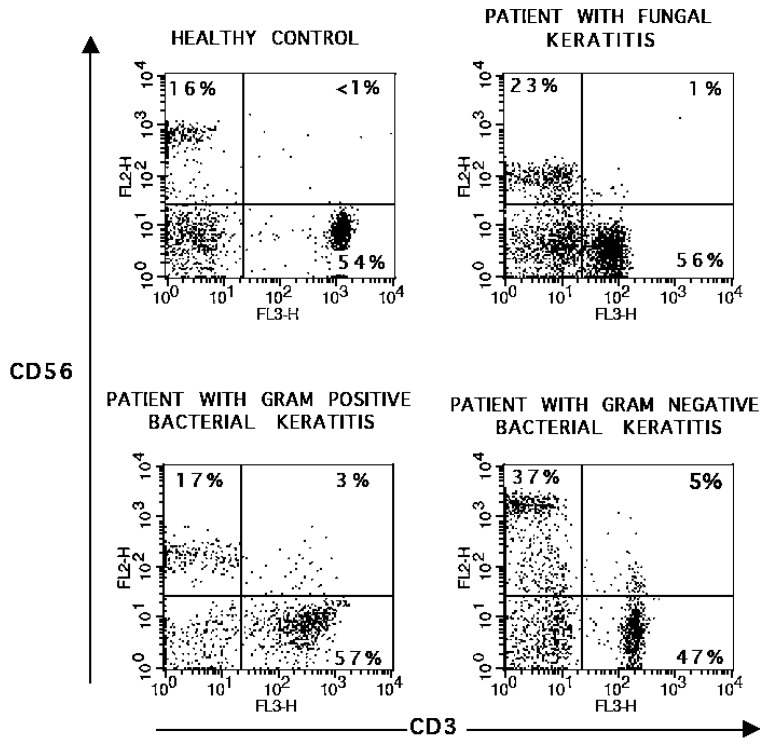
Frequency of CD3^−^CD56^+^ cells in peripheral blood mononuclear cells (PBMC), PBMC were stained with fluorescent-conjugated antibodies to CD3 and CD56 in a double immunofluorescence assay, as described in the Material and Methods section. Lymphocytes were gated by their physical properties (forward and side scatter) to analyze the staining of cell-surface markers. Data are presented as dot plots from gated lymphocytes. Representative images were taken from one patient of each group, and one of 48 healthy volunteers.

In order to know if peripheral blood mononuclear cells (PBMC) were involved in immune response during MK, we evaluated the frequency of circulating CD3^+^, CD4^+^, CD8^+^, CD19^+^ and CD3^−^CD56^+^ cells obtained from patients with microbial keratitis and results were compared with healthy volunteers. We observed that CD3^−^CD56^+^ cells were 1.5 times increased in patients with gram-negative bacterial keratitis, when were compared with healthy volunteers (*p* < 0.05). (Figure 2) After polyclonal stimulation, we observed 2.1 times diminished frequency of CD8^+^IFN-g^+^ T cells from gram-negative bacterial keratitis patients when compared with healthy volunteers (*p* < 0.05). All groups of patients were unable to induce CD8^+^IL-4^+^ T cells after polyclonal stimuli when were compared with healthy donors (*p* < 0.05) (Table 3).

### 2.4. IL-1β, IL-6, and IL-8 Are Increased in Tears Samples of Patients with Microbial Keratitis

**Figure 3:**
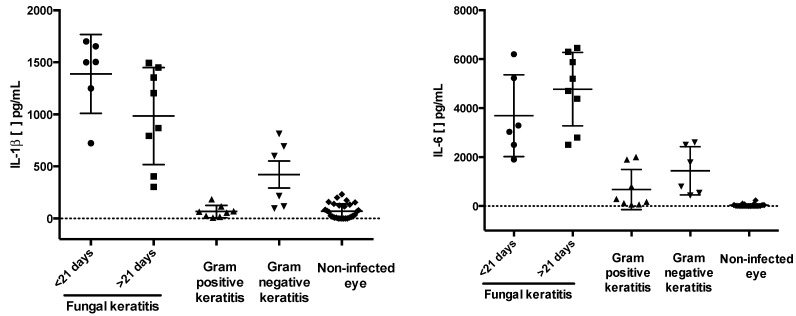

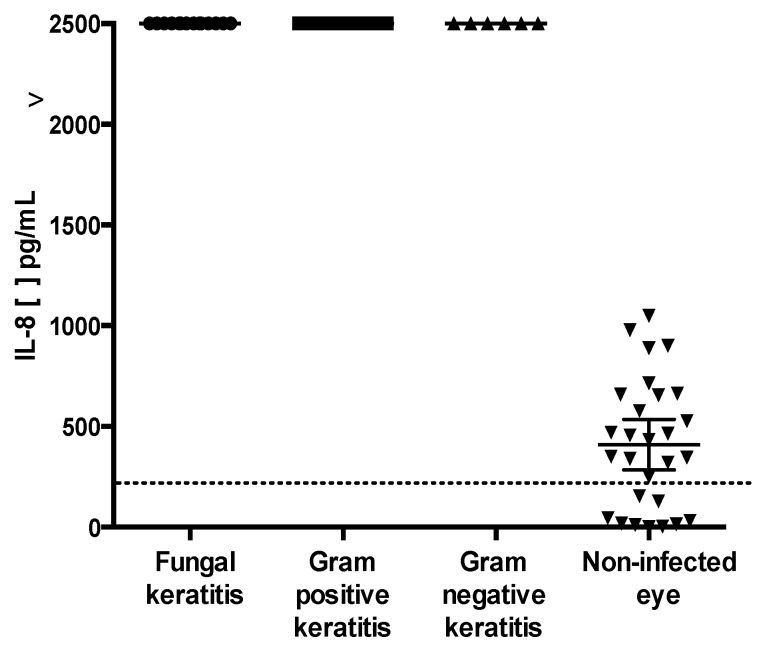
Tears cytokines in patients with microbial keratitis. The proinflammatory cytokines IL-1β, IL-6 and IL-8, were measured by cytometric bead arrays in tear samples from patients with microbial keratitis. Significant differences were observed in IL-1β, IL-6 and IL-8 in patients with fungal and gram-negative keratitis when compared with their contralateral non-infected eyes. IL-8 was significantly increased in all types of microbial keratitis versus the contralateral non-infected eye. Fungal keratitis presentation is showed as acute (<21 days) vs. chronic (>21 days). See Table 1, Table 2 and Table 3 for additional clinical data. The kit detection limits were as follows: IL-1β, 7.2 pg/mL; IL-6, 2.5 pg/mL; and IL-8, 3.8 pg/mL. In all groups, IL-8 levels were above the limit of detection.

To establish the potential involvement of cytokines in MK, we assessed the levels of secreted cytokines IL-1β , IL-6, IL-8, IL-10, IL-12p70, and TNF-α in tears samples obtained from infected eyes from patients with MK, tears samples obtained from contralateral non-infected eyes were used as controls. The concentrations of tear cytokines in patients with microbial keratitis are listed in Table 4. IL-1β, IL-6 and IL-8 were importantly detected. IL-1β measurements were as follows: 1135 ± 764 pg/mL in fungal keratitis; 423 ± 240 pg/mL in bacterial gram-negative keratitis, and 64 ± 40 pg/mL in bacterial gram-positive keratitis. IL-6 measurements were as follows: 4172 ± 1873 pg/mL in fungal keratitis, 1596 ± 971 pg/mL in gram-negative bacterial keratitis, 758 ± 1166 pg/mL in gram-positive bacterial keratitis. In all tears samples from patients with microbial keratitis, the concentration of IL-8 exceeded 2500 pg/mL (the limit detection of IL-8) (Figure 3).

**Table 3:** Frequency of PBMC sub-populations in patients with microbial keratitis.

PBMC Subpopulations	Fungal Keratitis (*n* = 14)	Gram-Positive Bacterial Keratitis (*n* = 8)	Gram-Negative Bacterial Keratitis (*n* = 6)	Healthy Volunteers (*n* = 48)	*p* Value
Percentage of CD3^+^ cells in PBMC	57 ± 10	55 ± 8	63 ± 9	62 + 11	NS between any groups
Percentage CD4^+^ cells in PBMC	34 ± 10	30 ± 3	31 ± 10	40 ± 13	NS between any groups
Percentage of CD8^+^ cells in PBMC	25 ± 11	24 ± 10	26 ± 10	28 ± 12	NS between any groups
Percentage of CD19^+^ cells in PBMC	12 ± 8	12 ± 9	10.1 ± 5	12 ± 7	NS between any groups
Percentage of CD3^−^ CD56^+^ cells in PBMC	28 ± 20	27 ± 13	39 ± 21 *	25 ± 12 *	*p* < 0.05
CD4^+^IFN-γ^+^ cells (After PMA/ionomicyn stimulation)	12 ± 10	16 ± 6	11 ± 6	10 ± 5	NS between any groups
CD4^+^IL-4^+^ (After PMA/ionomicyn stimulation)	1 ± 1	3 ± 3	2 ± 2	4 ± 2	NS between any groups
CD8^+^IFN-γ^+^ (After PMA/ionomicyn stimulation)	30 ± 27	16 ± 6 *	31 ± 23	34 ± 20 *	*p* < 0.05
CD8^+^IL-4^+^ (After PMA/ionomicyn stimulation)	1 ± 2 *	1 ± 1 *	2 ± 2 *	10 ± 7 *	*p* < 0.05, when compared with healthy volunteers

NS—Not significant; Mean ± SD (Standard deviation); * Significant differences when compared with healthy volunteers.

**Table 4:** Tear cytokines in microbial keratitis.

Tear Cytokine	Fungal Keratitis	Gram-Positive Bacterial Keratitis	Gram-Negative Bacterial Keratitis	Contralateral Non-Infected Eye	*p* Value (Compared with Contralateral Non-Infected Eye)
IL-1β pg/mL	1135 ± 764 *	64 ± 40	423 ± 240 *	58 ± 89 *	<0.05
IL-6 pg/mL	4172 ± 1873 *	758 ± 1166	1596 ± 971 *	25 ± 17 *	<0.05
IL-8 pg/mL	>2500 *	>2500 *	>2500 *	420 ± 377 *	<0.05
IL-10 pg/mL	43 ± 35	34 ± 23	107 ± 166	13 ± 3	NS
IL-12p70 pg/mL	12 ± 6	12 ± 1	4.0 ± 1	5 ± 5	NS
TNF-α pg/mL	28 ± 21	8 ± 4	8 ± 2	14 ± 7	NS

NS—Not significant; Results are expressed in pg/mL; Kit detection limits were as follows: IL-8, 3.8 pg/mL; IL-1β, 7.2 pg/mL; IL-6, 2.5 pg/mL; IL-10, 3.3 pg/mL; TNF-α, 3.7 pg/mL; and IL-12p70, 1.9 pg/mL.

## 3. Discussion

The cornea is a tissue highly resistant to microbial infection, but when the epithelium is damaged, pathogens are capable of invasion resulting in microbial keratitis. MK is a sight-threatening process, corneal infections progress rapidly, inducing epithelial defects and permanent ocular surface damage; Thus a prompt diagnosis of the causative agent is required to administer opportune treatments [1,2,3,4,5,6,7]. In animal models of MK, the immunological response is central to controlling infectious diseases of the eye and is related to the immunological mechanism of the corneal injury [13,14,15]. Two previous studies [16,17] have identified changes in tears cytokines from patients with MK, suggesting that proinflammatory cytokines are involved in fungal and bacterial keratitis. Less is known about changes in the systemic immunological response, together with the local immune response in patients with MK, prompting us to examine these topics here.

Since there are no pathognomonic signs of MK, clinical diagnosis is challenging, and the suspicion of the causative agent is based on the clinical presentation, *i.e.*, infiltrate border appearance, surrounding stromal haze, and presence or absence of hypopyon [18]. As other groups have reported [19,20,21] we did not observe statistically significant clinical data related with specific MK. Nevertheless, our findings suggest that the clinical pattern with regard to the size of the epithelial defect and stromal infiltration can be used as a medical clue to guide clinical diagnosis. Similarly, all patients included in this study had similar risk factors that were associated with the development of MK as reported by several authors [22,23,24].

In this work, we analyzed the frequency of lymphoid subpopulations in PBMC from patients with MK and observed that only patients with gram-negative keratitis had an increased percentage of circulating CD3^−^CD56^+^ NK cells. NK cells secrete cytokines in response to microbial products that stimulate various Toll-like receptors (TLRs), such as membrane-bound TLR4. TLR4 is receptor for LPS, a characteristically constituent of gram-negative bacteria, [25] and NK cells play a key role in polarizing a Th1 (IFN-γ) response on interaction with dendritic cells that have been exposed to microbial products in mucosal tissues [26]. Interestingly, studies on animal models of *Pseudomonas* keratitis have implicated NK and NKT cells in bacterial clearance and resistance to infection [27,28]. Further, sustained IFN-γ production accelerates corneal destruction and perforation [29], which is notable, since CD56^dim^ and CD56^bright^ are IFN-γ secreting cells, and CD56^bright^ cells produce abundant IFN-γ after continuous stimulation [30]. Although we did not analyze CD56 subsets due to the low number of patients with Gram-negative keratitis, all of them searched for ophthalmological assistance within one week after clinical presentation. (Table S3). Whether the increased frequency of CD3^−^CD56^+^ NK cells in PBMC observed in patients with gram-negative eye infections is involved with changes in CD56 cells subsets or the inflammatory eye response, is unknown and needs further investigation.

After polyclonal stimulation, we observed a diminished frequency of CD8^+^IFN-γ^+^ cells in patients with gram-negative bacterial keratitis. This finding could be related to the infectious process, since it suggests that patients with sepsis have an increased susceptibility to apoptosis of CD8 T cells, leading to an immunosuppressive state as the infection progresses [31]. However, more studies are needed to examine this possibility in patients with gram-negative bacterial keratitis.

IL-1β is a cytokine produced by mucosal epithelial cells of the ocular surface and immune cells, and its main function is to upregulate inflammation [32]. IL-6 is a strong inducer of acute-phase responses, promoting inflammation and the evolution of inflammatory states [33]. IL-8 is a CXC chemokine that mediates angiogenesis and the recruitment and activation of circulating leukocytes [34]. In this work, we observed an increase in these three cytokines in tears from patients with MK. Similarly, Vasanthi *et al.* [16] reported higher levels of IL-8 and IL-6 in tears from patients with fungal keratitis. After specific treatment, they found lower levels of proinflammatory cytokines in the tears and fewer polymorphonuclear (PNM) cells over the ocular surface. Interestingly, they observed that IL-8 remained slightly elevated, even after the ulcer healed, indicating continuous stimulation of the ocular surface. Our results are also consistent with Karthikeyan *et al*. [35], who demonstrated increased expression of mRNA to IL-8 and presence of PMN in the corneas from patients within 1-week infection with *Aspergillus flavus* and *Fusarium solani*.

Yamaguchi *et al*. [17] studied human bacterial keratitis, and observed a significant correlation between dendritic cells (DC) infiltration in the corneal stroma, and increased IL-1β, IL-6 and IL-8 levels in tears. In the same study, they observed lower cytokine concentration with less DC infiltration after instauration of treatment. It is possible that IL-1β, IL-6, and IL-8 synergize to induce immune-corneal damage in both, Gram-negative and gram-positive bacteria-infected patients, as suggested in the current study. Results of Vasanti *et al.* [16], and Yamaguchi *et al*. [17] suggest that proinflammatory cytokines levels change over the time: Rising in the initial stages of infection and declining in the last stages of infection or after treatment. This might explain the data dispersion observed in our study.

One limitation of the current work was that we included samples obtained from the contralateral non-infected eye as control tears. Recent evidence has suggested that pro-inflammatory cytokines are also increased in the contralateral eye [16,17]. Thus, the proper controls in future studies need to be tears from healthy donors without any inflammatory condition of the ocular surface.

The data shown here could lead to new perspectives in the treatment of severe human microbial keratitis; Wherein IL-1β, IL-6 and IL-8 are potential targets for biological therapy in patients with MK. *i.e.*, IL-6 blockage has been proposed for other IL-6-mediated ocular diseases, such as autoimmune uveitis, and in experimental corneal burns [36,37,38]. Based on their topical application and lower potential for side effects, the development of biological therapies might be readily adopted by ophthalmologists. Clinical trials are needed to examine their therapeutic value in patients with MK.

Our results implicate several immunological profiles apparently specific to the origin of keratitis: IL-8 > IL-6 in patients with bacterial keratitis; IL-8 > IL-6 > IL-1β and increased frequency of circulating CD3^−^CD56^+^ NK cells in patients with Gram-negative keratitis; while IL-8 = IL-6 > IL-1β in patients with fungal keratitis. Decisions with regard to therapeutic intervention should consider biologic therapies, in addition to antibiotic or antifungal treatment, to reduce ocular damage in patients with microbial keratitis.

## 4. Material and Methods

### 4.1. Patients

Twenty-eight individuals with microbial keratitis were included. Diagnosis was based on the clinical history and microbiology tests. To determine differences in the frequencies of PBMC, 48 healthy volunteers were used as controls. All participants gave their informed consent for blood, smear, and ocular lavage sampling after written information was provided. Inclusion of patients in this study was as follows: As soon as patients with MK were detected, they were informed about the study, and if they consented to participate, tear sampling was taken. Then treatments (topical or systemic) were initiated or modified. As an ophthalmological reference center, patients included in this study were at different stages of clinical outcome, and in some cases clinical evolution was very long. Information about clinical characteristics of patients, time of sampling and topical treatment is included in the Tables S1–S3. No relevant systemic medication is reported in the current study. The Medical Investigation and Ethics Committees of the Institute of Ophthalmology “Conde de Valenciana Foundation” in Mexico City approved this study. (CC-04-2007, in February 2007)

### 4.2. Clinical Evaluation

A complete medical history was taken, and ocular clinical signs were systematically documented by biomicroscopy during an ophthalmic examination. Epithelial defects and stromal lesions were measured with a slit-lamp (Carl Zeiss, Meditec Inc., Dublin, CA, USA) with and without fluorescein staining, and expressed in millimeters; Hypopyon is reported here as the percentage of occupied area in the anterior chamber of the eye.

### 4.3. Monoclonal Antibodies and Reagents

Phycoerythrin-labeled (PE) mouse IgG monoclonal antibodies (mAbs) against human CD4, CD56, IL-4, and fluorescein isothiocyanate (FITC)-labeled antibodies against human CD8, CD19, and IFN-γ, were purchased from BD PharMingen (San Diego, CA, USA). Phycoerythrin-Cy5-labelled mAbs anti-CD3 was obtained from e-Biosciences (San Jose, CA, USA). Lymphoprep (Ficoll 1.077 density) was obtained from Nycomed Pharma (Nyegaard, Oslo, Norway). Saponin, brefeldin-A, RPMI-1640 culture medium, PMA, ionomycin, and salts were purchased from Sigma Chemical Co (St. Louis, MO, USA). Sodium pyruvate, l-glutamine, and 2-mercaptoethanol were obtained from Gibco BRL (Rockville, MD, USA). Fetal calf serum was purchased from HyClone Labs (Logan, TU, USA).

### 4.4. Peripheral Blood Mononuclear Cells

Whole heparinized peripheral blood was diluted 1:2 (*v*/*v*) in phosphate-buffered saline (PBS), pH 7.2. Peripheral blood mononuclear cells (PBMCs) were separated on a Ficoll density gradient by centrifugation at 1700 rpm for 30 min at room temperature. After centrifugation, the interface cells were collected, washed twice, and counted on a hemocytometer to assess the viability by trypan blue dye exclusion.

### 4.5. Immunofluorescence Staining of Cell Surface Markers

Double or triple-color staining was performed on PBMC by direct immunofluorescence, using FITC-, PE-mAb and/or phycoerythrin-Cy5-labeled mAbs against human CD3, CD4, CD8, CD19 and CD56. Briefly, 2 × 10^5^ cells were suspended in 20 μL PBS supplemented with 0.2% bovine serum albumin and 0.2% sodium azide (PBA) and incubated with fluorochrome-labeled mAb for 30 min at 4 °C. After incubation, the cells were washed twice with PBA, fixed with 1% *p*-formaldehyde and analyzed by flow cytometry.

### 4.6. Cell Cultures

PBMC were cultured in 24-well flat-bottom cell culture plates (Costar, Cambridge, MA, USA) at 2 × 10^5^ cells/well in RPMI-1640 medium supplemented with 1 mM sodium pyruvate, 2 mM l-glutamine, 50 mg/mL gentamicin and 0.5% heat-inactivated fetal calf serum, and incubated at 37 °C in a 5% CO_2_ humidified chamber. After 24 h the culture medium was removed, and fresh culture medium supplemented with 10% heat-inactivated fetal calf serum, PMA/ionomycin (5–0.2 mg/mL) and brefeldin-A (10 mg/mL) were added. Four hours later, the cells were harvested and processed to measure intracellular cytokines expression by flow cytometry.

### 4.7. Immunofluorescence Staining of Intracellular Cytokines

Stimulated or non-stimulated PBMC were washed with PBA and stained with fluorescently labeled mAbs against CD4 or CD8 for 30 min. After washing, the cells were fixed with 4% *p*-formaldehyde in PBS for 10 min at 4 °C. The cells were washed twice with PBS and permeabilized with saponin buffer (0.1% saponin and 10% BSA in PBS) by shaking gently for 10 min at room temperature. The cells were then incubated with PE-labeled mAbs against human IL-4, and FITC-labeled anti-human IFN-γ. In all cases isotype-matched Ig/FITC and Ig/PE controls were used. After 30 min, the cells were washed with PBS, fixed again with 1% *p*-formaldehyde and analyzed by flow cytometry.

### 4.8. Flow Cytometric Analysis

All cells were analyzed for marker expression by collecting 5000 events on a FACS Calibur flow cytometer (Becton Dickinson, CA, USA) and using CellQuest Pro software. To analyze cell surface marker staining, a gate was drawn around the lymphocyte population based on their physical properties (forward and side scatter). To analyze intracellular protein staining, positive and negative fluorescence staining were set manually based on the distribution of stained cells with the isotype controls. Control stains were performed using isotype-matched mAb of unrelated specificity that were labeled with FITC-, phycoerythrin-Cy5- or PE. The background staining was subtracted from experimental values.

### 4.9. Determination of Soluble Cytokines

Samples from the infected eye and contralateral non-infected eye were obtained from the ocular surface by addition of 20 μL of sterile saline solution and immediately recovered with a sterile capillary, then tear samples were processed for determination of soluble cytokines. IL-1β, IL-6, IL-8, IL-10, IL-12p70, and TNF-α (Human Inflammation Cytokine Kit, BD Biosciences, San Jose, CA, USA), were measured by cytometric bead arrays, according to manufacturer’s instructions (BD Biosciences) and analyzed by flow cytometry with BD Cytometric bead array software version 1.1.1. on a FACS Calibur flow cytometer (FACSCalibur™, BD Biosciences, Franklin Lakes, NJ, USA).

### 4.10. Statistical Analysis

Kruskal-Wallis ANOVA followed by Dunn’s post-hoc test was used to detect significant differences. The analysis was performed using GraphPad software V.6 (GraphPad Software Inc., La Jolla, CA, US), and differences were considered statistically significant when *p* values were less than 0.05.

## 5. Conclusions

Our findings clearly indicate distinctive cytokines profiles in the tears of patients with microbial keratitis. Patients with bacterial keratitis, Gram-positive and -negative, have a predominance of IL-8 over IL-6 in tears. Additionally, subjects with gram-negative keratitis have a higher frequency of circulating CD3^−^CD56^+^ NK cells and IL-1β in tears. Patients with fungal keratitis express IL-8, IL-6, and IL-1β in tears without changes in circulating cells. Our results demonstrate how microbial pathogens can modulate local and systemic immune responses and clinical appearance. Further clinical trials are needed to evaluate IL-6 blockage as a therapeutic strategy in severe microbial keratitis to improve clinical outcomes.

## Supplementary Materials

Supplementary materials can be found at http://www.mdpi.com/1422-0067/16/03/4850/s1.
