# Supplementary material for: Expression of IL-8, IL-6 and IL-1β in Tears as a Main Characteristic of the Immune Response in Human Microbial Keratitis
Source: Int J Mol Sci. 2015 Mar 3;16(3):4850–64. doi: 10.3390/ijms16034850 (PMC4394453; doi:10.3390/ijms16034850)
Supplement: Supplementary file 1 [file ijms-16-04850-s001.pdf]

Supplementary Information

Table S1. Demographics and relevant medical history in patients with Fungal keratitis.

**Patient**	**1**	**2**	**3**	**4**	**5**	**6**	**7**	**8**	**9**	**10**	**11**	**12**	**13**	**14**
**Age (years)**	41	58	18	75	61	78	36	56	43	18	61	37	40	61
**Sex**	M	M	M	M	F	F	M	M	F	F	M	M	M	M
**Professional Activity**	Biologist	Farmer	Steelworker	Farmer	Housekeeper	Housekeeper	Office Worker	Farmer	Housekeeper	Student	Farmer	Worker	Student	Farmer
**Clinical Systemic Background**	None	DM	None	None	DM	DM	None	None	None	None	None	None	None	DM
**Clinical Local Background**	None	Vegetal trauma	None	Vegetal Trauma	None	Eyelid Scar	Vegetal Trauma	None	None	Soft Monthly Contact Lens	Alcoholism	None	None	Vegetal Trauma
**Topical Treatment at Moment of Tear Sampling**	Moxifloxacin Natamicin	Steroid Chloramphenicol	None	Moxifloxacin	Moxifloxacin	Gatifloxacin	Nafazolin Neomicin Cephalosporin	None	None	None	Moxifloxacin	Tobramicin	Moxifloxacin	Moxifloxacion
**Number of Days at Presentation and Tear Sampling**	35	30	9	30	30	15	30	20	5	1	60	20	21	60
**Visual Acuity (logMAR)**	Light Perception	Light Perception	Light Perception	Hand Motion	Light Perception	2.1	Hand Motion	Counting Fingers	0.2	Hand Motion	Light Perception	Hand Motion	Counting Fingers	Light Perception
**Eye Involved**	OD	OS	OD	OS	OS	OD	OD	OS	OS	OD	OD	OS	OD	OS
**Localization**	Irregular Central	Irregular Central	Central	Central	Ameboid Central	Central	Central	Para Central	Peripheric	Dendritiform Central	Central	Central	Central	Central
**Culture Isolate**	*Cephalosporium*	*Fusarium* spp.	*Fusarium* spp.	*Curvularia*	*Aspergillus* spp.	*Candida* spp.	*Fusarium* spp.	*Fusarium* spp.	*Fusarium* spp.	*Fusarium* spp.	*Fusarium* spp.	*Trichoderma* spp.	*Fusarium* spp.	*Fusarium* spp.

OD—Right eye; OS—Left eye; M—Male; F—Female; DM— Diabetes Mellitus.

Table S2. Demographics and relevant medical history in patients with Gram-positive bacterial keratitis.

Patient	1	2	3	4	5	6	7	8
Age (years)	32	62	81	72	69	75	89	45
Sex	M	M	F	M	F	F	F	F
Professional Activity	Horse Keeper	Retired	Retired	Retired	House	House	Home	Retired
Clinical Systemic Background	None	DM	None	None	None	DM	Gastritis, RA	RA
Clinical Local Background	Congenital Glaucoma	None	Trauma	Herpes	Herpes	None	None	None
Topical Treatment at Moment of Tear Sampling	None	None	None	None	None	None	Moxifloxacin, Prednisolone	Moxifloxacin
Number of Days at Presentation and Tear Sampling	7	90	20	21	2	20	7	10
Visual Acuity (logMAR)	Light Perception	Hand Motion	Light Perception	Hand Motion	Hand Motion	Light Perception	Light Perception	Light Perception
Eye Involved	OS	OD	OD	OD	OD	OD	OD	OS
Localization	Central	Central	Central	Central Abscess	Central	Central	Central	Central
Culture Isolate	*S. pneumoniae*	*Micrococcus varians*	*Streptococcus pneumoniae*	*Streptococcus pneumoniae*	*S. epidermidis* and *S. pneumoniae*	*Streptococcus* spp.	*S. epidermidis* and *S. pneumoniae*	*Streptococcus* spp.

OD—Right eye; OS—Left eye; M—Male; F—Female; DM—Diabetes Mellitus; RA—Rheumatoid Arthritis.

Table S3. Demographics and relevant medical history in patients with Gram-negative bacterial keratitis.

**Patient**	**1**	**2**	**3**	**4**	**5**	**6**
Age (years)	56	18	35	57	78	63
Sex	M	M	M	F	F	M
Professional Activity	Retired	Student	Unemployed	Home	Home	Retired
Clinical Systemic Background	None	Drug user	Malnutrition	DM and Systemic Hypertension	Non-treated Rheumatoid Arthritis	DM
Clinical Local Background	Amniotic membrane	Disposable Contact lens	Lens wear Penetrating keratoplasty	None	None	Chronic Dacryocistitis
Topical Treatment at Moment of Tear Sampling	Moxifloxacin	None	None	None	Cholarmphenicol	Moxifloxacin
Number of Days at Presentation and Tear Sampling	6	7	7	7	4	7
Visual Acuity (logMAR)	Light Perception	Hand motion	Light Perception	Counting fingers	Light Perception	0.5
Eye Involved	OD	OD	OS	OS	OD	OS
Localization	Central	Central	Central	Central	Central	Inferior
Culture Isolate	*Klebsiella pneumoniae*	*Pseudomonas aeruginosa*	*Pseudomonas aeruginosa*	*Serratia marcescens*	*Pseudomonas aeruginosa*	*Klebsiella ozanae*

OD—Right eye; OS—Left eye; M—Male; F—Female; DM—Diabetes Mellitus.
